# Molecularly-imprinted polymer-base bulk optode for the determination of ivabradine hydrochloride in Procoralan®

**DOI:** 10.1039/d2ra02064e

**Published:** 2022-06-15

**Authors:** Fatehy M. Abdel-Haleem, Mahmoud S. Rizk, Menna M. El-Beshlawy

**Affiliations:** Chemistry Department, Faculty of Science, Cairo University Giza Egypt fatehy@sci.cu.edu.eg; Center for Hazards Mitigation, Environmental Studies and Research (CHMESR), Cairo University Giza Egypt; Department of Chemistry, Faculty of Women, Ain Shams University Cairo Egypt

## Abstract

IVH may be administered orally twice per day for treating heart failure, sinus rhythm, heart-related chest pain and angina pectoris, and its overdose may cause problems such as blurred vision, uncontrolled blood pressure, prolonged bradycardia, and others. A molecularly imprinted polymer-based bulk optode, miptode, was constructed for the determination of ivabradine hydrochloride (IVH) in its pharmaceutical preparation Procoralan®. The molecularly-imprinted polymers (MIPs) were prepared in different ratios, and MIP3 had the highest imprinting factor (1.6) as an ionophore in the miptode preparation. The miptode was prepared using weight ratios of 30% PVC polymer, 62% nitrophenyl octyl ether (NPOE) plasticizer, 6% MIP3 ionophore, 1% tetraphenyl borate derivative (TPB) ion-exchanger, and 1% ETH7075 chromoionophore; this miptode exhibited an absorbance increase at 530 nm in the concentration range of 10^−2^–10^−5^ M with a detection limit of 3.1 μM using Tris–HCl buffer of pH 7.2. The miptode was imaged using AFM, and showed the dissolution of all components except MIP particles which exhibited restricted solubility. However, the incorporation of MIP3 as an ionophore improved the selectivity coefficient over the interfering species that may exist in the pharmaceutical formulation to an extent that was not reported before; *e.g.* coefficients of IVH over sodium, magnesium, and glucose were improved by 5, 4 and 2 orders, when compared to the previous sensor that operated with the molecular interaction mechanism. The selectivity improvement in miptode is due to the Key-Lock fitting (host-guest molecular recognition) between the MIP particles and the template IVH molecule which is transduced with the ion-exchange process of the chromoionophore. The miptode has a response time of 1–2 minutes, and a reliable lifetime of two months. The miptode was applied successfully for the determination of IVH in the pharmaceutical preparation Procoralan® with recovery values of 89–99.8% with low standard deviations of <1.2.

## Abstract 2

IVH may be administered orally twice per day for treating heart failure, sinus rhythm, heart-related chest pain and angina pectoris; its overdose may cause problems as blurred vision, uncontrolled blood pressure, prolonged bradycardia, and others.

## Graphical abstract

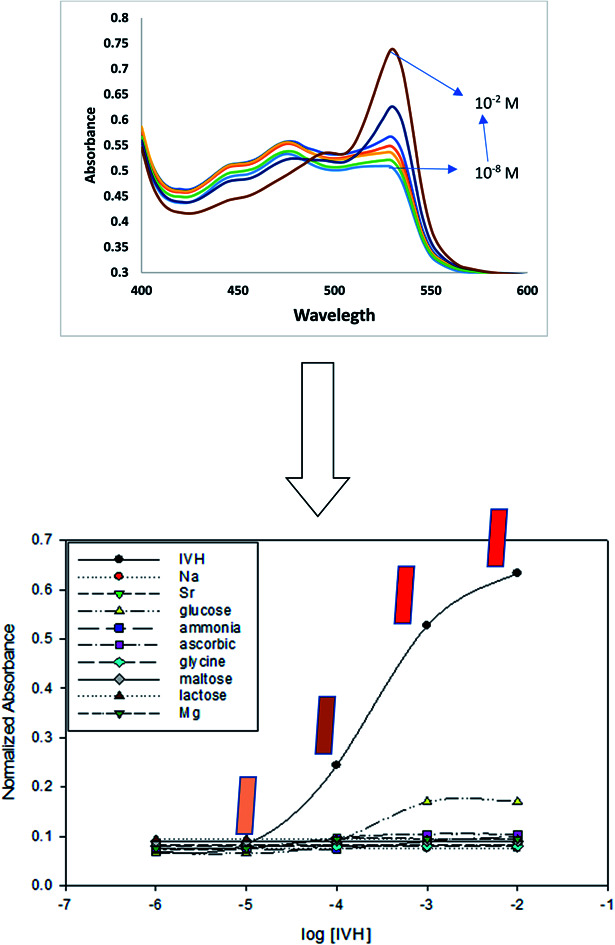

## 1. Introduction

Ivabradine HCl (IVH), named chemically as (S)-3-{3- [(3,4-dimethoxy bicycle [4.2.0] octa-1,3,5-triene-7-ylmethyl) methyl amino] propyl}-7,8-dimethoxy-2,3,4,5-tetrahydro-1*H*-3-benzazepine-2-one, is a drug for decreasing the heart rate through the selective inhibition of the pacemaker current for treating heart failure, sinus rhythm, heart-related chest pain and angina pectoris where there is no response from beta-blockers.^1^ IVH has been approved by the European Medicines Agency in 2005 and by the United States Food and Drug Administration in 2015; it is commonly used now as anti-ischemic and antianginal at stable angina,^1^ and may be administered twice daily by doses of 2.5, 5 or 7.5 mg per day.^2^ Because of the problems of IVH overdose (>15 mg per day) such as blurred vision, headache, uncontrolled blood pressure and prolonged bradycardia,^1^ different methods were reported for IVH quantification; these methods include spectrophotometry,^3,4^ chromatography,^5–10^ potentiometry,^3,11,12^ and spectrofluorimetry;^13^ these methods exhibited some disadvantages such as the high cost, the consumption of time and being of low selectivity.

Bulk optodes are among the most powerful analytical techniques that could be used for determination of different analytes.^14,15^ This wide spread of optodes is due to their numerous characteristics including the low detection limit, pH cross-sensitivity which enables variable linear dynamic range (LDR), determination of species of different charges, absence of the internal filling solution which permit miniaturization, besides their simplicity in preparation, cost effectiveness, applicability in routine analysis, and many others.^15,16^ Optodes were applied for the determination of cationic,^17^ anionic^17,18^ and neutral species;^17,18^ it was applied in environmental, medical, industrial and other applications with several improvement in the substrate materials and sensing schemes.^17–19^

During the last two decades, molecularly imprinted polymers (MIPs), compared to the non-imprinted polymers (NIPs), have grasped attention due to their variable applications in many fields due to their excellent advantages, including their high selectivity and affinity to their target template species, high mechanical and chemical stability, inertness, and being insoluble in water and most organic solvents.^20,21^ In addition, they can be easily prepared, cost effectiveness and applicability in harsh chemical media, temperature or pressure.^21^ The fitness between the template and the cavity of the MIP improves the different types of interactions of hydrogen bonding, van der Waals, covalent and ionic bonding, which results in high selectivity of the MIP over other macro-sized molecules.

**Fig. 1:**
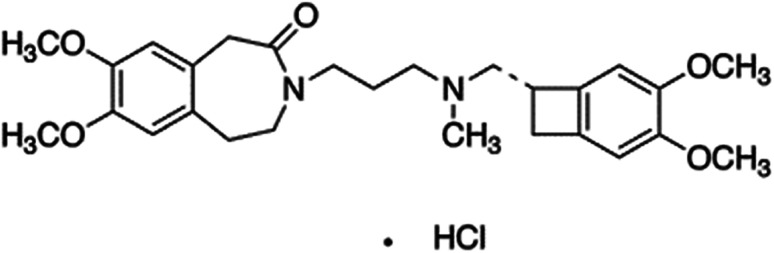
Structure of ivabradine hydrochoride.

In this work, merging between MIPs (or NIP) and optodes to form miptode (or niptode), was performed to combine the advantages of both to be used for several applications. We report here on the use of miptode impregnated with chromoionophore for the determination of IVH (Fig. 1) in its pharmaceutical preparation Procoralan®, which exhibited very interesting advantages which was not reported before by the ionophore-based optodes or electrodes.

## 2. Experimental

### 2.1. Materials

Ivabradine hydrochloride drug (IVH) and its pharmaceutical formulations Procoralan® (5 mg) were obtained from National Organization for Drug Control and Research (NODCR) (Giza, Egypt), and the local market, respectively. 2,2-azo-bisisobutyronitrile (AIBN, 98.0%, Sigma-Aldrich), methacrylic acid (MAA, 98.5%, Sigma-Aldrich), acrylamide (AC, 97.0%, Sigma-Aldrich), ethylene glycol dimthacrylate (EGDMA, 97.0%, Sigma-Aldrich), dimethyl sulfoxide (DMSO, 96.0%, Sigma-Aldrich), and *o*-nitrophenyl octyl ether (NPOE, 99.0%, Sigma-Aldrich) were used for the preparation of miptode and niptode. Hydrochloric acid (HCl, 30.0%, ADWIC), glucose (C_6_H_12_O_6_, 99.0%, ADWIC), maltose (C_12_H_22_O11, 98.5%, ADWIC), lactose (C_12_H_22_O_11_, 99.0%, ADWIC), glycine (C_2_H_5_NO_2_, 99.0%, ADWIC), sodium acetate (C_2_H_3_NaO_2_, 98.0%, ADWIC), sodium hydroxide (NaOH, 95.0%, ADWIC), Tris-(hydroxymethyl) methylamine (Tris, C_4_H_11_NO_3_, 95.0%, ThermoFischer), phosphoric acid (H_3_PO_4_, 98.0%, ADWIC), strontium chloride (SrCl_2_, 99.0%, ADWIC), sodium chloride (NaCl, 99.0%, ADWIC), magnesium chloride (MgCl_2_, 98.5%, ADWIC), anhydrous calcium chloride (CaCl_2_, 97.5%, ADWIC), ammonium chloride (NH_4_Cl, 99.0%, ADWIC) and potassium chloride (KCl, 99.0%, ADWIC) were used for preparing buffer solutions, standard solutions, and interfering ion solutions.

### 2.2. Preparation of the imprinted and non-imprinted polymers as ionophores

Molecularly imprinted (MIP) and non-imprinted polymers (NIP) of IVH were prepared and investigated using different characterization techniques as reported earlier.^22^ Briefly, different ratios of the monomer (MAA), the cross-linker (EGDMA) and the template (IVH) were mixed in a screw-capped Pyrex tube containing 2 mL DMSO, followed by the gradual addition of the initiator (AIBN); the mixture was degassed with a stream of pure Argon for 5 min and left in an oil-bath at 60 °C for 24 hours. A mortar and pestle was used for the fine powdering of the produced polymer white solid, then sieving to <45 μm size.^22,23^ IVH was extracted from the polymer by dispersing its particles in a mixture of (9 : 1, v/v) methanol-acetic acid for one day, filtering and dispersing in methanol and deionized water for one day. The IVH removal was confirmed spectrophotometrically at 286 nm. The non-imprinted polymer (NIP) was prepared systematically without IVH addition.^22^ The MIP and NIP was then dried their imprinting factor studies (IF) were performed. The MIP of the highest IF and its counterpart NIP were chosen as ionophores for the preparation of miptode and niptode.

### 2.3. Preparation of solution

Doubly distilled water was used through this work. Different buffers were prepared and tested; phosphate buffers (pH 5.1 and 8.0) and tris buffer (pH 7.2) were prepared by the addition of concentrated hydrochloric acid to 0.05 M NaOH and 0.05 M tris salt until the desired pH value.^12^ For preparation of 0.01 M IVH, the exact amount was weighed, dissolved in the least amount of buffer for complete dissolution, and completed with distilled water to the desired volume; the more diluted IVH solutions (10^−2^–10^−6^ M) were prepared by the appropriate dilutions.^12^

Preparing 10^−2^ M of the interfering ions was performed using the suitable buffer by weighing the exact amount and dissolving in the buffer; the more diluted concentrations were prepared using the same buffer by the appropriate dilutions of the stock 10^−2^ M of the respective interfering ion.^12^

### 2.4. Preparation of miptode and niptode

**Scheme 1:**
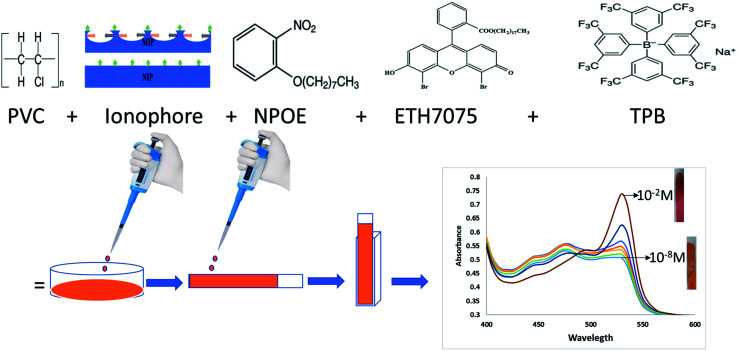
Steps of the preparation of the miptode to get the absorbance spectra.

For miptode (or niptode) preparation, the exact amounts in Table 1 were weighed, mixed and dissolved in a Petri-dish containing 3 mL tetrahydrofuran (THF), with stirring for obtaining reliable homogeneity. Onto a dust-free quartz slides (0.9 × 4 cm^2^, 1 mm thickness), the cocktail suspension was cast; it was left for drying for 20 minutes to form miptode (or niptode), Scheme 1.

**Table 1:** Composition of the different miptode, niptode, and ion-exchanger-based optode

No.	PVC	NPOE	Ionophore	ETH7075	TPB	Conc. range, M	Detection limit, M
Miptode 1	0.032	0.063	0.003 MIP3	0.001	0.001	10^−4^–10^−5^	1.0 × 10^−5^
Miptode 2	0.030	0.062	0.006 MIP3	0.001	0.001	10^−2^–10^−5^	3.1 × 10^−6^
Niptode 3	0.030	0.062	0.006 NIP3	0.001	0.001	10^−2^–10^−5^	5.0 × 10^−6^
IE-optode 4	0.032	0.066	—	0.001	0.001	10^−3^–10^−4^	8.0 × 10^−5^

The measurements were performed in absorbance mode using the miptode or niptode in a quartz cuvette (1 × 1 × 4 cm^3^) containing the analyte solution. IVH test solutions were prepared using different buffers. Miptode and niptode were conditioned in the selected buffer or solution for certain time before measurement. The normalized absorbance, the absorbance of the test solution divided by the background electrolyte,^24^ was plotted against the IVH concentration logarithm, Scheme 1.

### 2.5. Effect of pH

Optode technique is characterised by its pH cross sensitivity, which means the change of the linear dynamic range of concentration range (LDR) by the change of test solution pH due to the presence of pH-dependent chromoionophore;^15^ so, using buffer is a restriction in optode measurements. Different buffers were tested; phosphate buffers of pH 5 and 8. Also, tris buffer of pH 7.2 was tested. The buffer which exhibited wide LDR and low detection limit was chosen for the subsequent studies.

### 2.6. Selectivity

The selectivity of the miptode was measured using separate solution method,.^15,24^ The calibration curves for the different ions (IVH, glucose, maltose, sucrose, lactose, glycine, ammonia, sodium, strontium, magnesium) using the miptode was constructed by plotting the normalized absorbance *versus* the ion concentration logarithm. For confirming the selectivity, the mixed solution method was applied for the miptode and niptode as reported by wang and Meyerhof;^24^ 10^−2^ M interfering ion solution was added to 10^−4^ M IVH solution, and the shift in absorbance was recorded.

### 2.7. Reversibility and regeneration

Reversibility means measurements of the different concentrations from low to high concentrations, followed by measurements in the opposite direction; this study show the effect of the memory effect which give idea about characteristics of previously measured solutions.^15^ Regeneration of the optode means soaking of electrode in buffer or an acid to restore its state of equilibrium before the previous measurements.

### 2.8. Response and lifetimes

Response signal of the miptode was recorded at different times, and the stability of the signal was monitored. Lifetime was measured by performing measurements using miptode to get the calibration curve, and the change in LDR and detection limit was recorded.

### 2.9. Repeatability and reproducibility

Repeatability is the precision obtained when all measurements are made by the same analyst during a single period of laboratory work, using the same solutions and equipment, where reproducibility is the precision obtained under any other set of conditions.^25^ Repeatability is presented by showing error bars in the calibration curve, where reproducibility was tested by monitoring sensor-to-sensor variation.

### 2.10. Applications

For the preparation of application solutions, 5 tablets of Procoralan® were grounded, finely powdered, weighed exactly for preparing 10^−2^ M IVH solution, dissolved in the tris buffer, and filtered using a syringe filter; 10^−3^, 10^−4^ and 10^−5^ M IVH solutions were prepared by the proper dilutions;^26^ these solutions were measured using the miptode and the results were compared to that obtained by a reference high-performance liquid chromatography (HPLC) method.^10^

Also, buffer samples containing 2.5 mL urine were spiked with different amounts of standard IVH, the absorbance was measured using miptode 2, and the calibration curve method were applied for obtaining concentration of the solution and the recovery was calculated.

## 3. Results and discussion

### 3.1. Preparation of polymers and binding studies

Different ratios of crosslinker, monomer with/without template were performed for preparing MIP/NIP, respectively;^11^ MIP3 of template:monomer:cross-linker ratio of 1 : 6 : 40 showed best imprinting factor of 1.6, which was confirmed by the study of the binding isotherm and Scatchard plot.^11^ Accordingly, MIP3 and its counterpart NIP3 were used as recognition materials or ionophores for the preparation of the miptode and niptode.

### 3.2. Effect of miptode and niptode composition

In miptodes 1 and 2, different amounts of MIP were tested with the same amounts of the chromoionophore ETH7075, NPOE as a plasticizer, TPB as the ion-exchanger, and PVC as the polymer, Table 1; the higher amount of MIP in miptode 2 than miptode 1 caused an increase in LDR to three orders of magnitude, with a decrease in the detection limit to 3.1 μM, Table 1. An extra amount of MIP cannot be used because of the low solubility of MIP particles in PVC/THF membrane cocktail which may cause problems in the physical state of the miptode.^22,23^

**Fig. 2:**
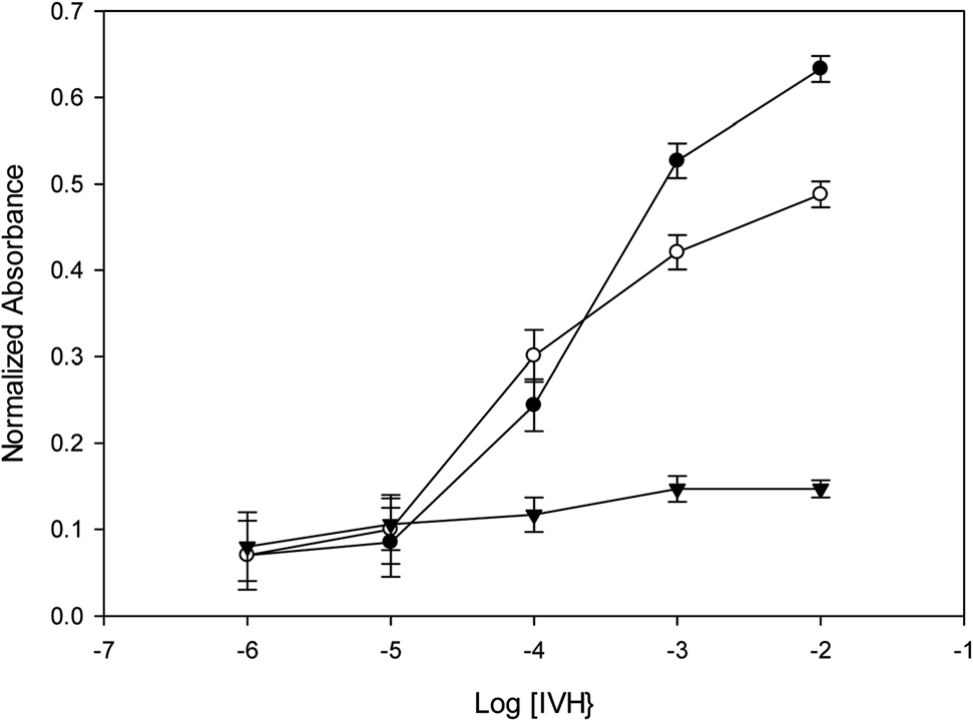
Calibration curves of the different sensors with error bars for three different measurements; (●) miptode 2, (○) nitpode 3, (▼) optode 4.

Incorporation of NIP as an ionophore in niptode 3, instead of MIP, resulted in the deterioration of the response signal with an increase of the detection limit and the decrease of the absorbance change with concentration, Fig. 2; this confirms the strength of the host–guest complexation between the MIP particles and IVH in miptode 2. For comparison purpose, IE-optode 4 based on the ion-exchanger TPB without ionophore was performed and tested for measurement of different IVH concentrations. IE-Optode 4 exhibited narrow LDR of 10^−3^–10^−4^ M with higher detection limit of 8.0 × 10^−5^ M; it can be interpreted by the absence of the recognition element, and the dependence on the ion-exchange process that is related to the p*K*_a_ of the chromoionophore and pH of the medium.^15^ So, miptode 2 exhibited the best response in terms of LDR, low detection limit and highest absorbance change with concentration change, Fig. 2.

**Fig. 3:**
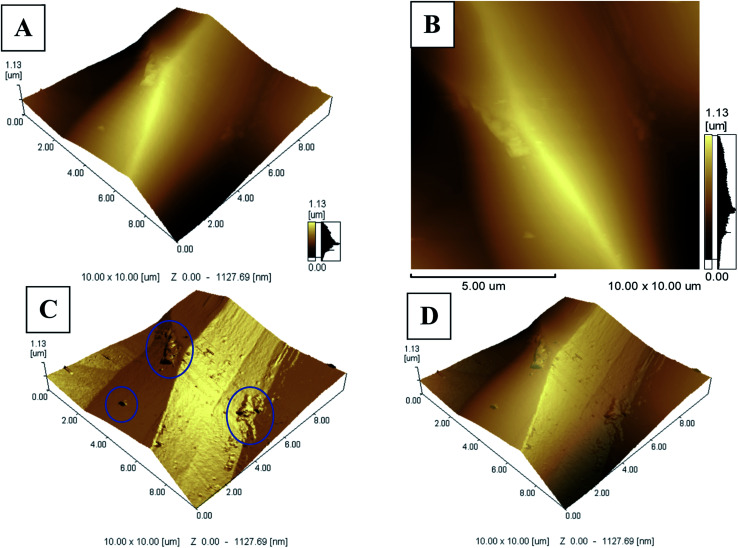
AFM images of miptode 2; (A and B) planar images, and (C and D) 3D images.

In order to check for the homogeneity and miscibility of the different components within the optode, AFM images were taken for miptode 2 as in Fig. 3. The planar image for the miptode 2 in Fig. 3A and B exhibited areas with different colours. In the same way, the 3D images of miptode 2 in Fig. 3C clarify the presence of some agglomerations of insoluble MIP particles (blue circle in 3C) which confirm the low solubility of MIP3 particles within membrane, and disturbed homogeneity. Accordingly, higher amounts of MIP particles cannot be added, which may affect the physical properties of the miptode membrane.

Although the limited solubility of the MIP particles within membrane cocktail, the reproducibility of miptode 2 was confirmed by the low values of the standard deviation as shown by small values of error bars in Fig. 2.

### 3.3. Response mechanism

**Fig. 4:**
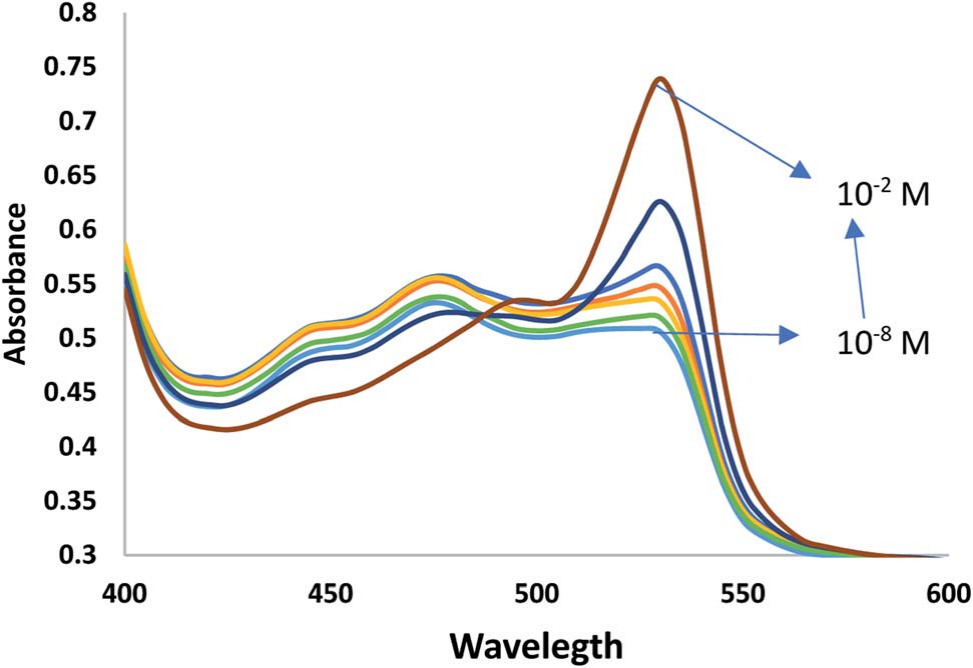
Spectra of miptode 2 in different IVH concentrations.

IVH has two pka values at 2.4 and 8.5, where its hydrochloride form exists as monocation at 7.2 using Tris–HCl buffer;^3,27^ the MIP particles can be considered as a neutral ionophore;^23^ IVH can be detected spectrophotometrically in presence of acidic chromoionphore ETH 7075 with an ionic additive to maintain electroneutrality within the membrane. The MIP particles is responsible for the selective binding of the analyte molecules with concomitant de-protonation of the chromionophore ETH7075, which leads to the increase of the absorbance at 530 nm and its decrease at 470 nm; the isosbestic point at 485 nm confirm the reversibility of protonation–deprotonation of the chromoionophore, Fig. 4.^23^ The sensing mechanism can be represented by the equations:

1 R^−^_m_ + L_m_+ IVH^+^_a_ → R^−^_m_ + LIVH^+^_m_

2 CH_m_ → C^−^_m_ + H^+^_a_

Where CH_m_ and C^−^_m_ represent the protonated and deprotonated forms of the chromoionophore ETH7075 in the membrane phase, L_m_ and LIVH^+^_m_ represent the neutral MIP3 particles in the free and complexed states, R^−^_m_ is the cation exchanger tetraphenyl borate in the membrane phase, and H^+^_a_ and IVH^+^_a_ represent the proton and the IVH cation in the aqueous solution.

### 3.4. Effect of pH

Using pH chromoionophores in the optode preparation facilitates the change in the LDR of application by changing the chromoionophore (p*K*a) or changing pH of measurement; this is known as pH cross sensitivity which is one of the most important issues of optodes. Miptode 2 was tested at different pH values, Table 2.

**Table 2:** Effect of pH on miptode2 response

pH-Buffer	LDR, M	DL, M
5.1 Phosphate	10^−3^–10^−5^	1.0 × 10^−5^
7.2 Tris–HCl	10^−2^–10^−5^	3.1 × 10^−6^
8.0 Phosphate	10^−3^–10^−5^	1.0 × 10^−5^

pH 7.2 exhibited the best results in terms of wide LDR and low detection limit; shift in pH to higher or lower values caused deterioration in these response properties; this is explained by the highest solubility in the physiological pH (38.9 mg mL^−1^) and the decrease in the solubility to 7.8 mg mL^−1^ at higher pH values, and decrease to 10.4 mg mL^−1^ at lower pH;^27^ this causes decrease in the amount of the free ions. Also, IVH has two basic pKa values of 2.4 and 8.5, and so at pH 7.2 it will exist as mono cation with maximum solubility.^27^ Accordingly, pH 7.2 using tris–HCl buffer was used for the further studies.

### 3.5. Selectivity

**Fig. 5:**
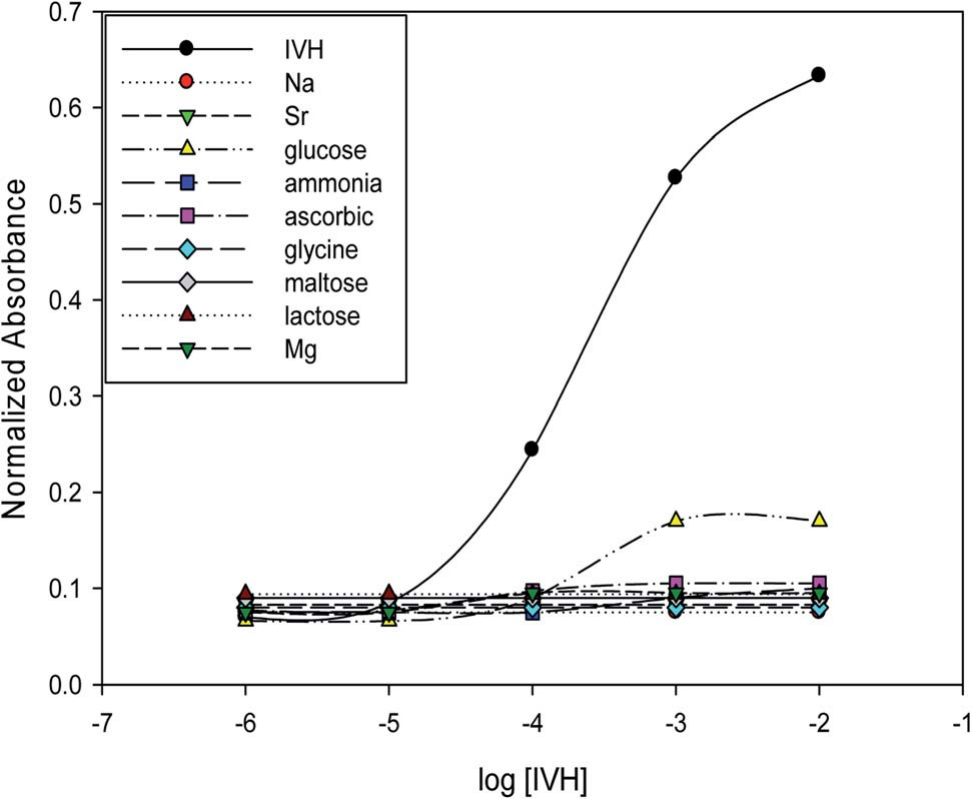
Selectivity and response of miptode 2 for IVH and different interfering ions applying separate solution method.

The selectivity of optode can be measured by several methods, as separate solution method (SSM) and mixed solution method (MSM).^15,24^ In SSM, the measurements were performed for each of the analyte and interfering ion separately using miptode 2; it exhibited very high selectivity towards IVH, as shown in Fig. 5; this is due to the suitability of the cavity within the tailored polymer for the template analyte IVH molecule.

Also, the MIP can form hydrogen bonding with ammonia and glucose as shown by the small response to miptode 2 in Fig. 4.^11,28^

**Fig. 6:**
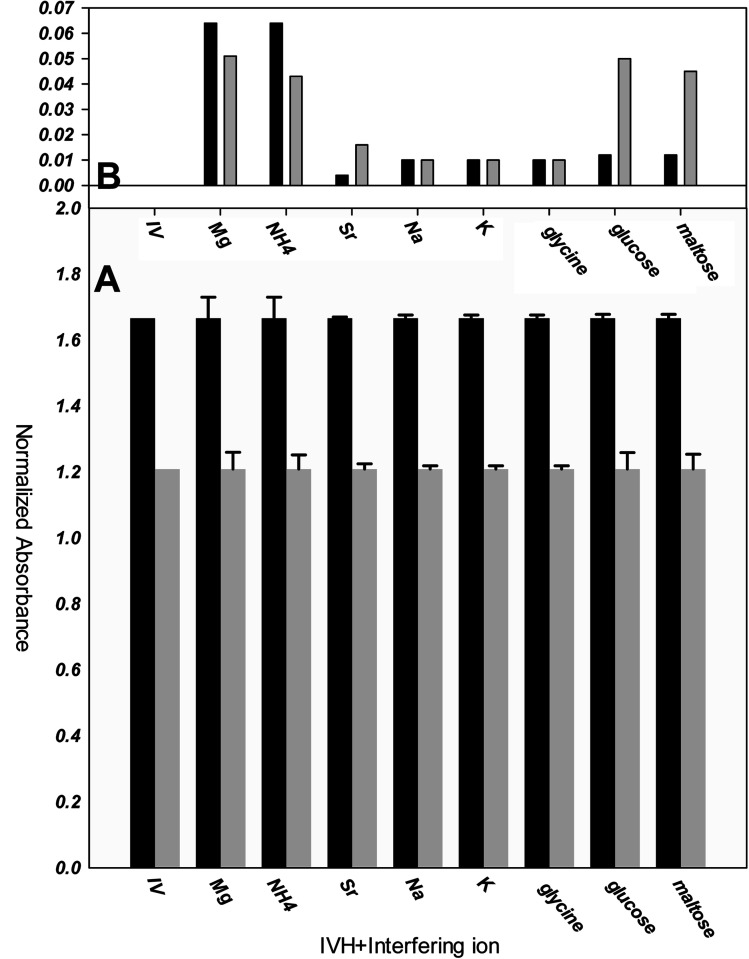
Selectivity of miptode 2 (black columns) and niptode 3 (grey columns) for IVH in the presence of different interfering ions using mixed solution method; (A) absolute value of the normalized absorbance of 10^−4^ M IVH+ 10^−2^ M interfering ion; (B) error bars of Fig. A that represent change in absorbance is more illustrated as columns.

To confirm the selectivity shown by SSM, measurements were performed by MSM reported by Wang and Meyerhoff;^24^ selectivity pattern of miptode 2 and niptode 3 were comparable for ionic species by applying the MSM as shown in Fig. 6. Also, it can be seen that glucose and maltose can interfere with niptode 3 due to their ability to form hydrogen bonding on the surface of NIP particles, but the molecular recognition as main sensing scheme for miptode 2 retard the possibility of this interference. These results confirm the high selectivity of miptode 2 over niptode 3, especially in case of hydrogen bonding forming species.^20,23^

By comparing the miptode 2 results to that of the CX-based potentiometric previously reported sensor,^12^ selectivity coefficients were improved by 2–5 orders of magnitude, Table 3. The highest selectivity obtained for miptode 2 is due to the host-guest interaction between MIP and template drug that is controlled by the mixed hydrogen bonding concomitant with ion exchange process between IVH^+^ and the proton of the dye. The deprotonation that is accompanied with hydrogen bonding results in strong signal. In case of other neutral molecules, hydrogen bonding only takes place between MIP and interfering ion with lower probability of deprotonation which lower the measured signal.^18,23^ This is the most important interesting property of MIP-based optodes.

**Table 3:** Selectivity coefficients of miptode 2 compared to the previously reported methods that depends on molecular recognition, as measured by separate solution method

	Miptode	MIP-ref. 11	CX-ref. 12
Na	< −6.0	−2.6	−1.79
Sr	< −6.0	—	—
Glucose	−4.2	−1.8	−1.21
Ammonia	−5.0	−2.1	−2.00
Ascorbic acid	< −6.0	−1.10	−0.82
Glycine	< −6.0	−1.79	−1.18
Maltose	< −6.0	−1.80	−1.20
Lactose	< −6.0	−1.77	−1.17
Mg	< −6.0	−2.54	−2.17

Bakker *et al.*^15^ reported that existence of higher amount of ionophore in case of optodes will improve the selectivity; the wt% in case of miptode 2 in this work, MIP-based ion selective electrode^11^ and CX-based ion selective electrode^12^ are 6%, 3% and 1%, respectively. Also, Bakker compared the selectivity coefficients for ISEs and optodes with the same amount of ionophore and ion-exchanger, which showed improved of the selectivity coefficient by and order of magnitude at exchanger amount of 10.0 mmol Kg^−1^; in this work ion-exchanger is added with amount of 13.4 mmol Kg^−1^ in comparison to absence of ion-exchanger in previous work, which resulted in the improved selectivity coefficients by values of 2–4 orders of magnitude, especially in case of neutral and divalent interfering species.^15^

### 3.6. Reversibility and regeneration

Reversibility was tested between concentrations of 10^−4^, 10^−3^ and 10^−2^ M IVH from low to high concentrations with repeating the measurement cycle. Fig. A shows that about 90% of the signal was recovered when moving between measurements. This deterioration in response may be due to the leaching of some of the miptode components (chromoionophore, ion-exchanger) to the measurement solution which resulted in signal decrease, and also due to memory effect which means the existence of the effect of last signal of the miptode.^15^ This deterioration in response and the memory effect can be overcome by soaking of the miptode in buffer for long time of 2 hours or for about 5 minutes in 2 M HCl.^16^ Applications of other types of polymers such as polyurethane instead of PVC may also account for this problem;^15^ polyurethane may be used without plasticizer, and even in presence of plasticizer, dissolution to the measurement solution is of low probability.

### 3.7. Response time and lifetime

**Fig. 7:**
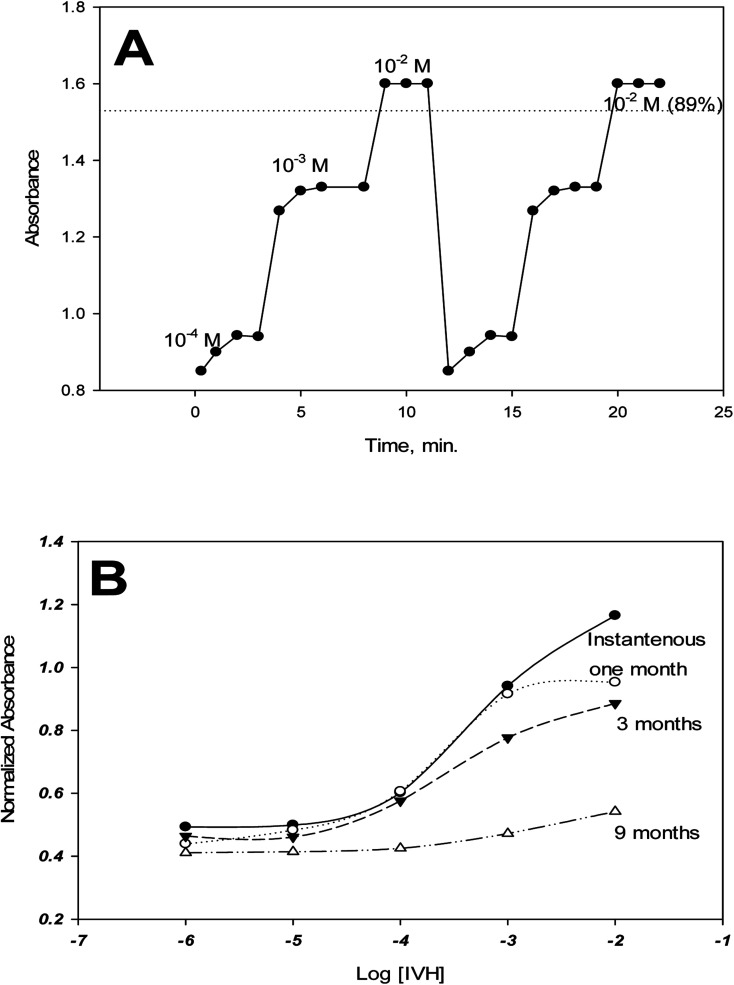
Reversibility and response time (A), and life time (B) of the miptode.2.

The response time is defined as the time of measurement at which the sensor exhibits 90% of its stable signal.^15,29^ As shown in Fig. 7A, the response time of miptode 2 is about 2 minutes for low concentrations and one minute for high concentrations.

The life time of miptode 2 was recorded by performing measurements of calibration curve periodically, which exhibited that miptode 2 was stuck very well for quartz plate; the same miptode can be used for two months, where the same cocktail in the Petri-dish can be used for more than ten months when kept in dark, with minimum change in the LDR and detection limit, and some change in the response, Fig. 7B. Although the response of miptode 2 decreased to large extent, it could be used for determination of IVH successfully; the sensor can be used for more than 2 months, and it just need to be soaked in 2 M HCl for 5 minutes followed by soaking in buffer for 20 minutes. Generally, lifetime of 2 months is very safe to use the miptode with minimum change in its characteristics.

### 3.8. Repeatability and reproducibility

**Fig. 8:**
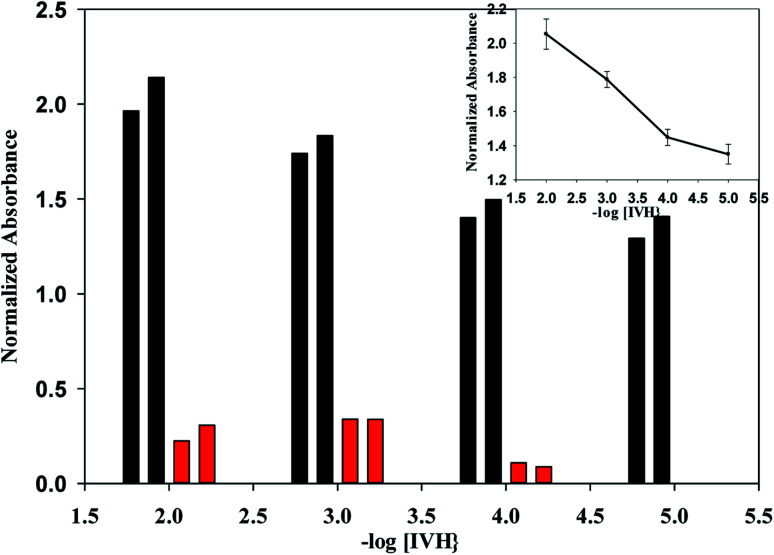
Sensor-to-sensor variation of miptode 2, (black columns) the absolute values of normalized absorbance, (red columns) the change in normalized absorbance when move from low to higher concentration, inset is the calibration curve with error bars for two different sensors.

Repeatability was confirmed by using the same miptode sensor for many times at the same day, and very good results was obtained as shown by error bars that confirms the low values of standard deviation, Fig. 2. Also, sensor-to-sensor variation (or reproducibility) was tested using different sensors at the same day and at different days, and the results was reasonable in terms of low standard deviation, as confirmed in Fig. 8.

### 3.9. Applications

Different samples of Procoralan® that cover this application range were measured with miptode 2 and the results were compared to that obtained by reference HPLC method.^10^ Also, spiked urine samples containing standard IVH were determined by calibration curve method using miptode 2 (Table 4).

**Table 4:** Determination of different amounts (mg L^−1^) of Procoralan® and of standard IVH in spiked urine samples using miptode s and compared to a reference method^10^.^a^

Miptode 2	HPLC^10^	%Recovery	Miptode 2	Theoretical	%Recovery
4.98	5.08	98.00 ± 1.2	4.50	5.050	89.10 ± 0.9
50.13	52.87	94.80 ± 0.8	47.20	50.50	93.46 ± 0.7
505.37	540.90	93.40 ± 0.2	504.50	505.00	99.80 ± 0.2

a It can be seen from results in Table 4 that recovery values are ranged between 89.1 and 99.8 with very low values of standard deviations (<1.2) of three measurements; these high recovery and low standard deviation values show the accuracy and precision, respectively, of the proposed miptode, which confirm the applicability of the miptode.

## 4. Conclusion

A MIP-based bulk optode, miptode, was built and used to determine Ivabradine hydrochloride. The mechanism for the recommended Miptode was the same as for ionophore-based optodes, which respond to simple cations with simultaneous deprotonation of the chromoionophore ETH7075, resulting in absorbance increase at 530 nm. In terms of lowering the detection limit, pH cross sensitivity proved advantageous. The miptode demonstrated highly promising selectivity results over most lipophilic species. Miptode 2 was successfully employed for the determination of various samples of Procoralan® that span the application range and the results were compared to that obtained by reference HPLC technique.

## Conflicts of interest

There are no conflicts to declare.

## Supplementary Material
